# Development of a novel high quantum efficiency MV x‐ray detector for image‐guided radiotherapy: A feasibility study

**DOI:** 10.1002/mp.13900

**Published:** 2019-11-04

**Authors:** Jian Liu, Yuan Xu, Aram Teymurazyan, Zisis Papandreou, Geordi Pang

**Affiliations:** ^1^ Department of Physics Ryerson University Toronto Ontario M5B 2K3 Canada; ^2^ Department of Physics University of Regina Regina Saskatchewan S4S 0A2 Canada; ^3^ Odette Cancer Centre Department of Radiation Oncology University of Toronto Toronto Ontario M4N 3M5 Canada; ^4^ Present address: Radiation Medicine Northwell Health Lake Success NY 11042 USA

**Keywords:** EPID, MV x‐ray imaging, portal imaging, scintillating fibers

## Abstract

**Purpose:**

To develop a new scintillating fiber‐based electronic portal imaging device (EPID) with a high quantum efficiency (QE) while preserving an adequate spatial resolution.

**Methods:**

Two prototypes were built: one with a single pixel readout and the other with an active matrix flat‐panel imager (AMFPI) for readout. The energy conversion layer of both prototypes was made of scintillating fiber layers interleaved with corrugated lead sheets to form a honeycomb pattern. The scintillating fibers have a diameter of 1 mm and the distance between the centers of neighboring fibers on the same layer is 1.35 mm. The layers have 1.22 mm spacing between them. The energy conversion layer has a thickness of 2 cm. The modulation transfer function (MTF), antiscatter properties and sensitivity of the detector with a single pixel readout were measured using a 6‐MV beam on a LINAC machine. In addition, a Monte Carlo simulation was conducted to calculate the zero‐frequency detective quantum efficiency (DQE(0)) of the proposed detector with an active matrix flat‐panel imager for readout.

**Results:**

The DQE(0) of the proposed detector can be 11.5%, which is about an order of magnitude higher than that of current EPIDs. The frequency of 50% modulation ($f_{50}$) of the measured MTF is 0.2 mm${}^{-1}$ at 6 MV, which is comparable to that of video‐based EPIDs. The scatter to primary ratio (SPR) measured with the detector at 10 cm air gap and 20 × 20 cm${}^{2}$ field size is approximately 30% lower than that of ionization chamber–based detectors with a comparable QE. The detector noise which includes the x‐ray quantum noise and absorption noise is much larger than the electronic noise per pixel of the flat‐panel imager at a dose of less than two LINAC pulses. Thus, the proposed detector is quantum noise limited down to very low doses (∼a couple of radiation pulses of the LINAC). A proof‐of‐concept image has been obtained using a 6‐MV beam.

**Conclusions:**

This work indicates that by using scintillating fibers and lead layers it is possible to increase the thickness of the detecting materials, and therefore the QE or the DQE(0) of the detector, while maintaining an adequate spatial resolution for MV x‐ray imaging. Due to the use of lead as the spacing material, the new detector also has antiscatter property, which will help improve the signal‐to‐noise ratio of the images. Further investigation to optimize the design of the detector and achieve a better combination of DQE and spatial resolution is warranted.

## 1 Introduction

Most of the current megavoltage (MV) electronic portal imaging devices (EPIDs) used in radiotherapy rooms are based on the indirect detection of x rays where a copper plate is coupled with a scintillator screen typically made of terbium doped gadolinium oxysulfide (Gd2O2S:Tb).^1^ The copper plate serves two purposes: it blocks the low‐energy scattered x rays and secondary electrons from the patient and interacts with the primary x rays to produce energetic secondary electrons, which then travel into the scintillator screen and generate optical signals in visible spectrum. The visible photons are then detected by a pixelated array of photodiodes of an active matrix flat‐panel imager (AMFPI) to form an image.^2^ Due to the lateral spread of the light in the scintillator, the scintillator layer has to be thin in order to maintain a good spatial resolution. As a result, the detective quantum efficiency (DQE) of most EPIDs used in clinics is limited to only 1–2% or less,^3^ resulting in an inefficient use of x rays and thus hindering the wide use of MV cone beam CT for image‐guided radiotherapy (IGRT).

Various approaches have been explored to improve the DQE while maintaining the spatial resolution of the MV x‐ray detectors, such as using pixelated scintillator arrays^4^, ^5^, ^6^, ^7^, ^8^, ^9^, ^10^, ^11^, ^12^, ^13^, ^14^, ^15^ and building multilayer imagers.^1^, ^16^, ^17^, ^18^, ^19^ A variety of segmented scintillators composed of noncrystalline materials such as Gd2O2S:Tb powder^13^ and crystalline materials such as thallium‐doped cesium iodide,^4^, ^5^, ^10^ bismuth germanate^11^, ^12^, and cadmium tungstate^9^, ^14^, ^15^ have been investigated to optimize the tradeoff between DQE and spatial resolution. However, the cost of crystalline scintillator is high and it is complex to fabricate pixelated crystalline arrays without defects.^13^

Another approach involves the use of a stack of four conventional MV x‐ray detection layers.^1^, ^16^, ^17^, ^18^, ^19^ By combining signals from all four layers, a zero‐frequency DQE of about 6.7% was observed and the SNR was increased by a factor of 1.7.^1^ A limitation of this approach might be the degradation in modulation transfer function (MTF) if more layers are used to further increase the DQE, due to the defocusing caused by beam divergence.

Pang et al. proposed to use Cerenkov radiation (subsequently referred to as Cerenkov portal imaging detector or CPID) generated in optical fibers to form an image.^20^, ^21^, ^22^, ^23^ Optical photons produced in the fiber core and within the acceptance angle of the fiber are then guided to the detecting device by total internal reflection, the detector can be made thicker to increase the quantum efficiency (QE) and at the same time maintain acceptable spatial resolution. It was discovered that the CPID is an inherent antiscatter detector for MV x‐ray imaging, due to the fact that scattered x rays have lower energy and are less likely to generate secondary electrons with high enough energy to produce Cerenkov radiation.^22^ However, it was found that a large area (e.g., 40 × 40 cm${}^{2}$) CPID would require a technology (i.e., an AMFPI with an avalanche gain) that is not yet available because of the low light yield of Cerenkov radiation. Plastic scintillating fibers were proposed to replace the nonscintillating fibers to overcome the weaknesses of the CPID, and the proposed scintillating fiber EPID can also be used for dose measurement because of its water‐equivalent property.^24^

Recently Blake et al.^25^ built a prototype EPID with plastic‐scintillating fibers for simultaneous imaging and dosimetry in radiotherapy. The fibers were coated with a layer of extramural absorber to prevent optical cross talk between fibers. Although light generated in the fiber will be guided along the fiber, x rays and energetic secondary electrons can still travel transversely between fibers and produce optical photons along their path resulting in degradation in spatial resolution. In this work, we propose to use lead as septa material between scintillating fibers. The lead not only can eliminate cross talk between fibers but also help convert primary x rays to secondary electrons to increase the QE and block scattered low‐energy x rays to improve spatial resolution. Two prototypes were made: one with a single pixel readout (hereinafter referred to as prototype scintillator) and the other with an AMFPI for readout (hereinafter referred to as prototype detector). The line spread function (LSF), antiscatter property, and sensitivity of the prototype scintillator were investigated. A proof‐of‐concept image was also obtained using the prototype detector. A Monte Carlo simulation was conducted to calculate the zero‐frequency detective quantum efficiency (DQE(0)) of the proposed detector.

## 2 Materials and Methods

**Figure 1:**
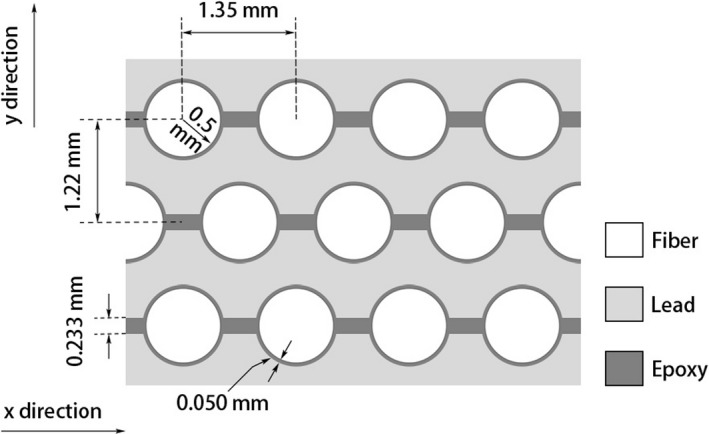
Diagram of the lead‐fiber scintillator matrix structure (top view).

**Figure 2:**
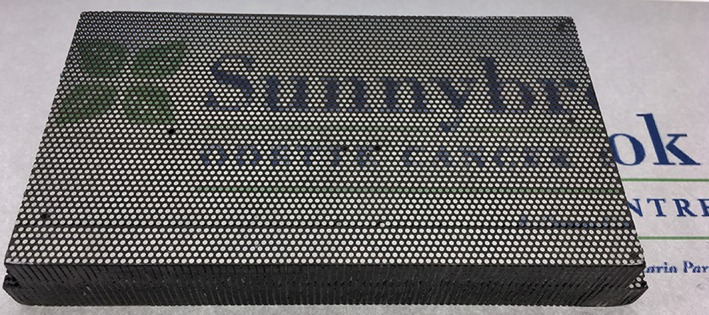
The detector block or the prototype scintillator (without image readout) placed on top of the “Sunnybrook — Odette Cancer Centre” logo. [Color figure can be viewed at http://www.wileyonlinelibrary.com]

The energy conversion layer used for both the prototype scintillator and the prototype detector is a lead‐fiber scintillator matrix which was made by interleaving scintillating fibers (Kuraray SCSF‐78MJ, Kuraray Co., Ltd., Tokyo, Japan) in lead to form a honeycomb pattern. Very thin corrugated lead sheets and scintillating fibers were bonded together using BC‐600 optical epoxy (Saint‐Gobain Crystals, OH, USA). A diagram of the lead‐fiber scintillator matrix (top view) is shown in Fig. 1 and a picture of the lead‐fiber scintillator matrix or the detector block (without image readout) on top of the “Sunnybrook — Odette Cancer Centre” logo is shown in Fig. 2.

The lead‐fiber matrix, constructed at the Department of Physics, University of Regina, was initially 4 m long and part of an early prototype of one of the 48 identical azimuthal segments in a barrel calorimeter for a GlueX experiment. This calorimeter, shaped as a 390‐cm‐long (hollow) cylinder having inner and outer radii of 65 and 90 cm, respectively, with a total weight of 28 tons, detects neutral and charged particles in the nuclear physics experiment GlueX,^26^ whose central goal is the mapping of hybrid and exotic mesons generated by the excitation of the gluonic field binding the quarks. A 12 × 7 × 2(thickness) cm${}^{3}$ piece was cut to be used as a proof‐of‐concept prototype detector described here.

Kuraray SCSF‐78MJ is a double‐clad fiber. The diameter of the fiber used in this study is 1 mm. Materials that make‐up the fiber and their properties are listed in Table 1. When the lead‐fiber scintillator matrix was first coupled with a flat‐panel imager for image readout, the signal was low, probably due to the lead contamination on the fiber ends introduced during the detector fabrication process as well as the fact that fiber ends were not polished after cutting.

**Table 1:** Material properties of the Kurary SCSF‐78MJ scintillating fiber.

	Material	Refractive index	Density (g/cm${}^{3}$)
Core	Polystyrene (PS)	1.59	1.05
Inner cladding	Polymethylmethacrylate (PMMA)	1.49	1.19
Outer cladding	Fluorinated polymer (FP)	1.42	1.43

To quantify the properties of the proposed detector, a prototype scintillator with a single pixel readout was built. A fiber at the center of the lead‐fiber scintillator matrix was removed and a longer fiber of the same type (Kuraray SCSF‐78MJ) was inserted into the scintillator matrix. The other end of the longer fiber was coupled with a 10‐m‐long optical fiber (P600‐10‐UV‐VIS, Ocean Optics, Inc. Dunedin, FL, USA) through a fiber optic dual switch (FOS‐2 × 2‐TTL, Ocean Optics, Inc. Dunedin, FL, USA). The optical fiber was connected to a photomultiplier tube (PMT) (R6095, Hamamatsu Photonics K.K., Japan) which was placed outside the treatment room to minimize the signal due to direct interaction between the radiation and the PMT. The output signal from PMT was read using an electrometer (Model 530, Victoreen, Cleveland, Ohio, USA). Measurements were carried out at the Odette Cancer Centre, Sunnybrook Health Sciences Centre in Toronto, using a 6 MV beam generated from a LINAC machine (Synergy, Elekta, UK). The LINAC was calibrated to deliver 1 cGy per monitor unit (MU) in water at a source‐to‐axis distance (SAD) of 100 cm and a depth of *d*_max_ = 1.5 cm at a field size of $10\;\times \;10\;{\text{cm}}^{2}$. All field sizes referred to in this work are defined at the iso‐center (ISO), which is the point where the rotational axes of the LINAC gantry, collimator, and treatment couch meet. The collimator angle was set to $0^{\circ }$. The dose referred to in the work is the dose to water at a depth of *d*_max_ = 1.5 cm with SAD of 100 cm setup.

### 2.A Scintillating fiber emission spectrum

Upon irradiation, scintillating fibers emit a spectrum of optical photons. The emission spectrum of the scintillating fiber (Kuraray SCSF‐78MJ) was measured using a FLAME‐T spectrometer (Ocean Optics, Inc., Largo, FL, USA) and the LINAC machine (at 6 MV). One end of the scintillating fiber was connected to the spectrometer. The rest of the scintillating fiber was rolled into a circular shape with a diameter of about 15 cm and placed on the solid water (GAMMEX RMI, Wisconsin, USA) which was, in turn, placed on the treatment couch. Then a 1.5‐cm‐thick solid water buildup was placed on top of the fiber. The fiber was kept at the ISO of the LINAC during the measurement. A black cloth was used to cover the fiber and all lights in the LINAC room were turned off to prevent ambient light from entering the fiber and being detected by the spectrometer.

### 2.B Linearity

**Figure 3:**
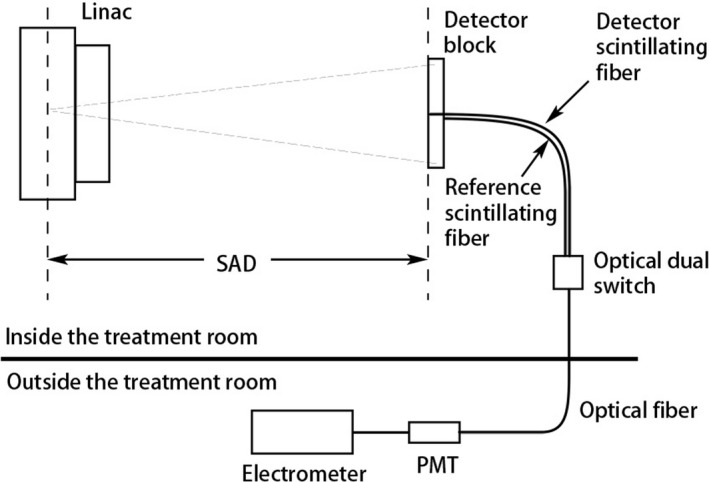
Experimental setup for linearity measurement of the prototype scintillator.

Ideally the response of the detector should increase linearly with radiation dose. To measure the linearity, another scintillating fiber (reference fiber) that was 2 cm shorter than the scintillating fiber (detector fiber) inserted into the prototype scintillator was running beside the detector fiber (Fig. 3). To get the signal from only the segment of the detector fiber in the detector, the signal difference, that is, the signal from the detector fiber minus the signal from the reference fiber, was measured. The radiation dose was varied in the range from 10 to 250 cGy to investigate the dependence of the output signal on the dose.

### 2.C X‐ray absorption efficiency

The x‐ray absorption efficiency (AE) in this work is defined as the percentage of x rays that interact with a detector, which equals the maximum achievable quantum efficiency (QE) of the detector. To measure the AE, the prototype scintillator without image readout or the detector block was placed on the couch at the ISO of the LINAC machine. An ionization chamber (Farmer model A19) was placed 50 cm away from the beam exit side of the detector to minimize the effect of scattered x rays generated in the detector block. Ionization chamber readings were recorded at a fixed exposure with and without the presence of the detector block in the 6 MV x‐ray beam. The field size at the ISO was varied from $2\;\times \;2\;{\text{cm}}^{2}$ to $6\;\times \;6\;{\text{cm}}^{2}$ to check if there is any dependence of measured values on x‐ray field size.

### 2.D Spatial resolution

**Figure 4:**
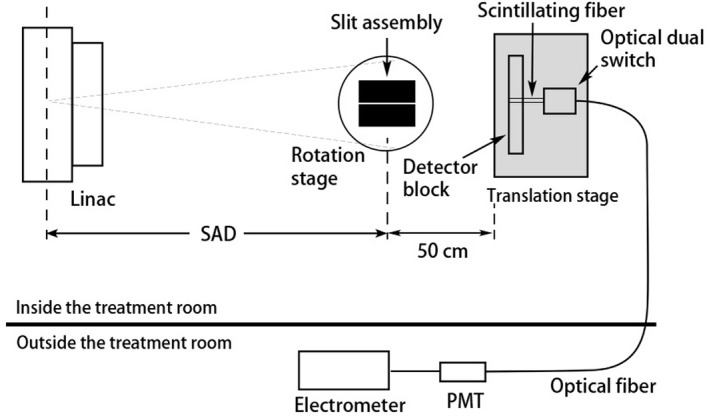
Experimental setup for line spread function measurement.

To characterize the spatial resolution of the prototype scintillator, MTF was obtained from the Fourier transform of the LSF. To measure the LSF, a slit beam was generated by passing the x‐ray beam through a slit made of two steel blocks, each with dimensions of 3.5 cm × 7 cm × 10.5 cm. The surfaces of 7 cm × 10.5 cm of two steel blocks were squeezed together with shims of 80 μm thickness in between. The slit assembly was placed on a rotation stage which was placed between the x‐ray source and the prototype (Fig. 4). The rotation stage can rotate at $1^{\circ }/300$ per step.

**Figure 5:**
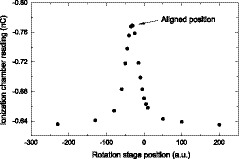
The ionization chamber reading is shown as a function of rotation stage position. The peak location indicates where the slit object in Fig. 4 is aligned to the incident x‐ray beam.

Before taking readings using the prototype, an ionization chamber (NE2571 Farmer chamber) was used to align the slit assembly with the incident x‐ray beam. To align the slit, ionization chamber readings were recorded for a given dose at different rotation stage angles. The alignment was achieved when ionization chamber reading was at the maximum (Fig. 5). The alignment was then checked with a radiochromic film attached to the beam exit surface of the slit. An exposure of 284 cGy produced a dark line on the radiochromic film.

The prototype scintillator was positioned on a translation stage with a precision of 0.01 mm/step and was placed 50 cm away from the slit object to minimize the scattered radiation from the slit object. The radiation field (at 6 MV) was set to $3\;\times \;3\;{\text{cm}}^{2}$ at the ISO to reduce the effect of scattered radiation from the LINAC head. The prototype scintillator was then moved to scan across the slit beam (from −15 to 15 mm) to obtain a raw LSF. To account for any residual signal from scattered radiation and leakage radiation through the 10.5‐cm‐thick steel blocks, a “no‐slit” scan was obtained when the slit assembly was rotated 2${}^{\circ }$ off alignment. The difference between the “slit” data (i.e., the raw LSF data) and the “no‐slit” data yielded the corrected LSF.

The corrected LSF was processed using the method described by Munro and Bouius.^27^ The LSF was first made symmetric with respect to the center of the LSF and then the tail of the LSF was fitted analytically before applying the Fourier transform. The modulus of the Fourier transform yielded the measured MTF after normalized to unity at zero frequency. The measurements were repeated on different days to estimate measurement uncertainties.

### 2.E Antiscatter property

**Figure 6:**
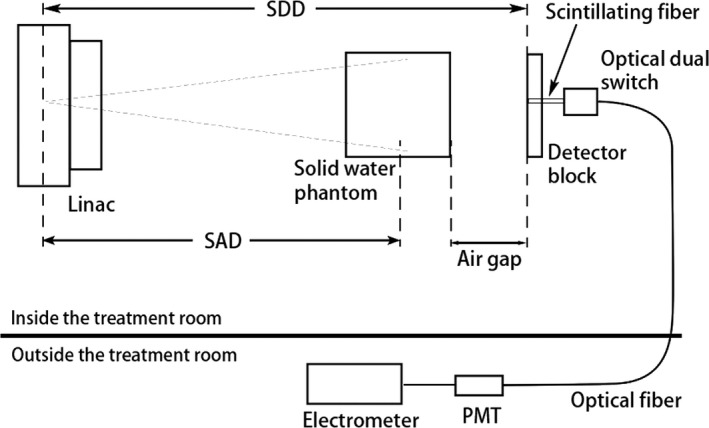
Experimental setup for measuring the antiscatter properties of the prototype scintillator.

To measure the SPR of the prototype scintillator, a $30\;\times \;30\;\times \;30\;{\text{cm}}^{3}$ solid‐water phantom was used to mimic the patient and generate scattered x rays (Fig. 6). The center of the solid water phantom was placed at the ISO. The air gap (AG) which is the distance between the x‐ray exit surface from the solid water and the prototype scintillator was varied from 5 to 75 cm. The light output from the scintillating fiber under irradiation from a 6‐MV beam for a given dose was measured using the PMT with a supply voltage of 500 V.

**Figure 7:**
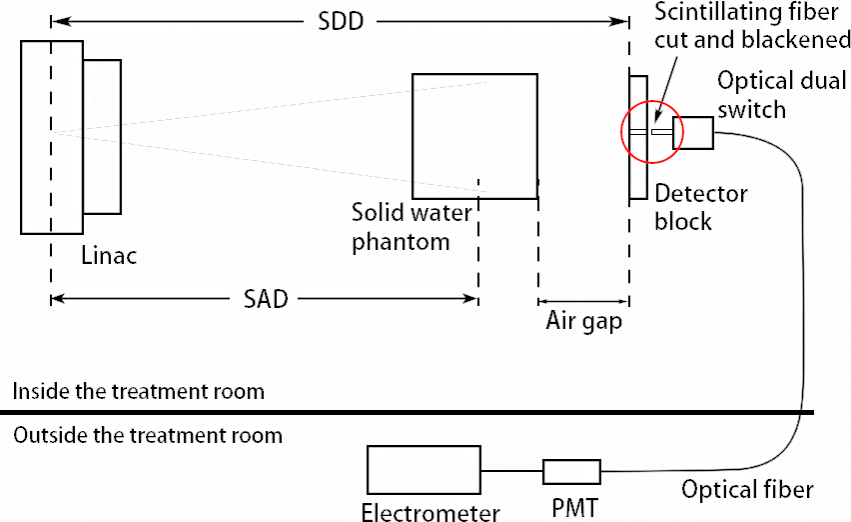
Experimental setup for measuring signal from the fiber segment outside the detector block. [Color figure can be viewed at http://www.wileyonlinelibrary.com]

The signal measured with the setup in Fig. 6 was generated from the scintillating fiber segment embedded in the detector block plus the scintillating fiber portion that connects to the optical switch. To get the signal only from the scintillating fiber segment in the detector block, the scintillating fiber outside the detector block was cut off and the end blackened (Fig. 7). The signal difference from measurements shown in Figs. 6 and 7 yielded the signal from the scintillating fiber segment in the detector block only. To calculate the SPR, the primary signal due to the interaction between primary x rays and the prototype scintillator has to be determined. As the measured signal is always the sum of the primary signal and the scatter signal for a finite field size, the primary signal is obtained by extrapolating the measured signal to zero field size at each AG, which was validated using Monte Carlo simulation in Tian and Pang.^28^ When the field size approaches zero, the measured signal approaches the primary signal.^28^ Primary signals were determined for measurements with and without the phantom placed between the x‐ray source and the prototype scintillator. The measurements were repeated three times to estimate the uncertainty of the extrapolated primary signal. Assuming that the total signal measured with solid water phantom at a given field size is *T*, then

(1) T=P+S,

where *P* and *S* are the primary and scatter signals, respectively, in the presence of the phantom. Similarly, in the absence of the phantom, we have

(2) $$T^{\prime }=P^{\prime }+S^{\prime },$$

where $T^{\prime }$ and $P^{\prime }$ are the total and primary signals without the phantom for the same LINAC output, that is, the same MUs as that used for measuring *T*, and $S^{\prime }$ is the scatter signal from the LINAC collimators and the prototype scintillator itself in the absence of the phantom.

**Figure 8:**
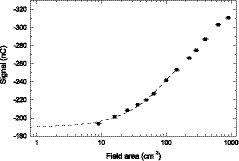
The detector signal is plotted as a function of field sizes for an air gap of 15 cm. The dashed line is the extrapolation to field size zero.

Figure 8 shows an example of data extrapolation to get the primary signal. To verify the extrapolated primary signal is correct, we investigated the dependence of *P* and $P^{\prime }$ on source to detector distance (*SDD*). For a thick detector, the dependence should be as follows^28^

(3) $$P\propto \frac{1}{SDD(SDD+L)},$$

where *L* is the thickness (2 cm) of the detector. Furthermore, we also calculated the ratio of *P* to $P^{\prime }$ (denoted as $R_{0}$) and checked whether $R_{0}$ varies with the AG.

Once the primary signal *P* is obtained, the SPR can be calculated from the measurement for a given field size as

(4) $$\text{SPR}=\frac{T-P}{P}.$$

### 2.F Sensitivity of the detector

An optimum x‐ray imaging system should be quantum noise limited. To determine the sensitivity of the proposed detector at low imaging doses, the signal and the root‐mean‐square (rms) noise per pixel were measured with the prototype scintillator at a dose close to two LINAC pulses. Since the LINAC used for this experiment was not able to deliver just one or two LINAC pulses (0.026 cGy each) at a time, the slit object was used to attenuate the x‐ray beam to reduce the dose to the prototype. The experimental setup for sensitivity measurement is similar to the LSF measurement (Fig. 4). The slit object was first aligned to the incident beam and then rotated 4${}^{\circ }$ off alignment. The field size was set at $5\;\times \;5\;{\text{cm}}^{2}$. Then 1 MU was delivered and the electrometer reading recorded. The measurement was repeated 146 times. To determine the dose delivered after attenuation by the slit object, an ionization chamber was used to measure the dose with and without the slit object blocking the incident beam.

**Figure 9:**
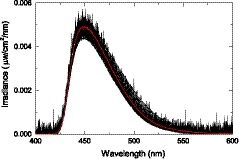
A scintillating fiber emission spectrum is shown with the fiber excited by a 6‐MV beam. The red line is the average of 78 measurements (black lines).

The signal $N_{\mathrm{ph}}$ per pixel of the detector is defined as the number of scintillating photons exiting the scintillating fiber, and can be calculated from the measured charge *Q* of the PMT as^20^

(5) $$N_{\mathrm{ph}}=\frac{Q}{e\eta G\gamma \xi },$$

where *e* is the electron charge, *η* is the average optical QE of the PMT in the emission band of the scintillating fiber (Fig. 9), *G* is the gain of the PMT, *γ* is the light transmission coefficient through the optical dual switch and the optical fiber, and *ξ* is the light transmission coefficient at the interface between the optical fiber and the PMT. *G* was determined to be $2.41\;\times \;10^{5}$ at 750 V — the PMT voltage used for the sensitivity measurement — from the PMT specification provided by the manufacturer. *γ* was introduced to account for the reflective light loss at the interface between the scintillating fiber and the optical dual switch and the light attenuation in the approximately 10‐m‐long optical fiber. The value of *γ* was measured to be 0.40. *ξ* accounts for the reflective light loss at the interface between the optical fiber and the PMT. It was estimated that *ξ* = 0.85 using the Fresnel equation at normal incidence.^20^ The average optical QE of the PMT, *η*, was calculated numerically using

(6) $$\eta =\int_{\lambda_{1}}^{\lambda_{2}}\eta \left(\lambda \right)\frac{dN(\lambda )}{d\lambda }d\lambda /\int_{\lambda_{1}}^{\lambda_{2}}\frac{dN(\lambda )}{d\lambda }d\lambda ,$$

where $\lambda_{1}\;=\;420$ nm and $\lambda_{2}\;=\;560$ nm, *η*(*λ*), obtained from the PMT product data sheet, is the optical QE of the PMT at wavelength *λ*, and *N*(*λ*) is the scintillating fiber emission spectrum (Fig. 9). The value of *η* was calculated as 0.18 for 420 nm < *λ* < 560 nm.

The rms noise of the optical signal per pixel is defined as^20^

(7) $$\sigma_{\mathrm{ph}}=\sqrt{\left\langle N_{\mathrm{ph}}^{2}\right\rangle -{\left\langle N_{\mathrm{ph}}\right\rangle }^{2}},$$

where 〈⋯〉 denotes the (arithmetic) average over a large number of samples.

According to the theory of noise transfer through a stochastic gain process^29^ and based on Eq. (5), we have the following relation between the rms noise of the optical signal per pixel ($\sigma_{\mathrm{ph}}^{2}$) and that of the charge signal of the PMT ($\sigma_{d}^{2}$)

(8) $$\frac{\sigma_{d}^{2}}{e^{2}}=G^{2}\left[{\eta^{\prime }}^{2}\sigma_{\mathrm{ph}}^{2}+\left\langle N_{\mathrm{ph}}\right\rangle \eta^{\prime }\left(1-\eta^{\prime }\right)\right]+\eta^{\prime }\left\langle N_{\mathrm{ph}}\right\rangle \sigma_{\mathrm{PMT}}^{2},$$

where $\eta^{\prime }\left(<\;1\right)$ and the rms noise of the charge signal of PMT, $\sigma_{d}$, are defined as

(9) $$\eta^{\prime }=\eta \gamma \xi ,$$

and

(10) $$\sigma_{d}=\sqrt{\left\langle Q^{2}\right\rangle -{\left\langle Q\right\rangle }^{2}}.$$

The quantity $\sigma_{\mathrm{PMT}}^{2}$ is the variance of the PMT gain and is given by^20^

(11) $$\sigma_{\mathrm{PMT}}^{2}=\frac{G^{2}}{\delta -1},$$

where *δ* = 3.09 is the average gain per stage at 750 V.

Based on Eqs. (5), (8), (9), (10), and (11), we can calculate the mean optical signal $\left\langle N_{\mathrm{ph}}\right\rangle$ and its rms noise $\sigma_{\mathrm{ph}}$ of the proposed detector from the measured 〈*Q*〉 and $\sigma_{d}$ of the PMT. In the above noise analysis, the LINAC output noise was neglected since it is small comparing to the PMT noise, accounting for only 1–2% of the total noise.^20^

### 2.G DQE(0)

The single pixel prototype used in the above experiments cannot be used to measure noise correlations between different pixels. In addition, it used a PMT instead of an AMFPI for signal readout. Therefore, an in‐house developed Geant4 (version 4.10.05.p0)‐based Monte Carlo simulation has been used to evaluate the DQE(0) of the proposed detector with an AMFPI for image readout. The full chain of x‐ray quantum absorption, x‐ray energy deposition in the detector, as well as scintillation light production and transport in fibers was simulated. The geometry of the detector as modelled in simulation was based on description provided in beginning of Section 2 (Fig. 1), but incorporated a surface area of $31.34\;\times \;28.32\;{\text{cm}}^{2}$ and coupled with an AMFPI for image readout.^30^ Due to the lack of chemical composition of some of the materials the BC‐600 epoxy, binding the plastic scintillator optical fibers and the corrugated lead layers, was modelled as polyvinyl pyrolidone.

The simulation accounts for electromagnetic interactions of x rays, electrons and positrons (Geant4 EM physics option 3) as well as for the generation and transport of optical photons within the scintillating optical fibers. For the transport of optical photons, our simulations assumed a smooth interface of dielectric surfaces between fiber core and cladding layers with refractive indices of 1.59 and 1.49, respectively. Optical photons, arriving at cladding–epoxy interface, are assumed to be completely attenuated. The bulk attenuation length (∼3.5 m) provided by the manufacturer was applied in order to model optical photon absorption in fiber cores.

Furthermore, to investigate how the DQE(0) is affected by the *optical coupling efficiency* between the AMFPI and the scintillating fibers in the detector we considered varying the *photon detection efficiency* (PDE) of the AMFPI from 100% to 0.2%. A 40% PDE, for example, means that only 40% of the optical photons emitted from bottom ends of the scintillating fibers are absorbed and converted to the image signal by AMFPI. In simulations a narrow ($1.0\;\times \;1.0\;{\text{mm}}^{2}$) beam of 6‐MV x rays was incident at the surface of the detector at the coordinates of one of the fibers. The x‐ray spectrum used in the simulation was generated by treatment planning system (Pinnacle3, Philips, Fitchburg) and described in Teymurazyan and Pang.^24^ The total number of optical photons reaching the bottom ends of scintillating fibers was integrated on an event‐by‐event basis to obtain the detector signal per pixel. The DQE(0) of the prototype detector was calculated as^31^

(12) $$DQE\left(0\right)=\frac{M_{1}^{2}}{M_{2}},$$

in which $M_{n}$ is the *n*‐th moment of the distribution of optical photons reaching the bottom ends of the optical fibers and available for detection by the AMFPI defined as

(13) $$M_{n}=\frac{1}{N_{x}}\sum_{m}m^{n}p\left(m\right),$$

where *p*(*m*) is the probability distribution function of *m* optical photons reaching the bottom ends of the fibers for a given x‐ray, and *N*_x_ is the number of incident x rays.

### 2.H Proof‐of‐concept image

To obtain a proof‐of‐concept image, we built a prototype detector by coupling a slightly large version of the lead‐fiber scintillator matrix (with an imaging area of $23\;\times \;13\;{\text{cm}}^{2}$) to a flat‐panel imager (Model RID 1640 AL2, PerkinElmer Optoelectronics, Fremont, CA, USA) for image readout. To this end, all the original layers above the sensor (a‐Si) in the flat‐panel detector had to be removed so that the senor could be exposed to the scintillating fibers of the prototype for image readout. These layers included a front plate (aluminum), a copper plate, a graphite sheet, and a Lanex Fast‐B screen (see Table 1 in Tast et al.^32^). Care was taken not to damage the sensor (and the glass substrate) in the panel during the process.

The removal of the graphite sheet (∼0.4 mm thick) was most challenging. The graphite sheet was attached to the flat panel by both a metal adhesive and black silicone. It sat about 1 mm from the sensor with the Lanex Fast‐B screen in between. To remove graphite sheet, the black silicone had to be removed first. This was done using a soft‐medium plastic scraper. After the silicone was removed, a scalpel was used to carve away at about $1\;\times \;1\;{\text{cm}}^{2}$ corner section of the graphite sheet. The adhesive and the graphite sheet were carved all the way down exposing the gap between the graphite sheet and the sensor. The graphite sheet was then gently pried off the metal adhesive.

Once all these layers were removed, the prototype detector was coupled with the flat‐panel sensor. Due to its weight, the prototype cannot sit directly on the very fragile glass sensor. To overcome this problem, an aluminum plate of 3.2 mm thickness with a rectangular hole of approximately the size of the prototype was made. The aluminum plate was placed on top of the flat panel with the prototype siting in the hole so that the bottom surface of the prototype was close to (with ∼1 mm air gap) but did not touch the flat‐panel sensor.

To acquire a MV x‐ray image the new prototype detector with the flat‐panel readout was placed on the floor of a LINAC room with the source to the detector surface distance of about 219 cm. A PIPSPro QC‐3V phantom^33^ was placed on top of the detector. Movie images were taken at 6 MV with a frame rate of 3.5 frames/s, which was not synchronized with the delivery of the LINAC pulses.^34^ Gain and offset corrections were completed prior to the image acquisition.

## 3 Results

### 3.A Scintillating fiber emission spectrum

Figure 9 shows the scintillating fiber emission spectrum in absolute irradiance excited by a 6‐MV beam at the gantry angle 0${}^{\circ }$. The red line is the average of 78 measurements (black lines). The fiber emits light in the band from 420 to 560 nm upon irradiation. The emission peak is at 450 nm, which is in the most sensitive region of the PMT used for this work. The emission peak of the scintillating fiber is well matched to the absorption band of the AMFPI fabricated from hydrogenated amorphous silicon (a–Si:H).^30^

Since each of the measured spectrum shown in Fig. 9 is the spectrum measured in a 6‐MV beam minus the background, the irradiance has negative values in the background region of the spectrum, that is, outside the fiber emission band. The negative values are not shown in Fig. 9. The average of many measurements in the wavelength range outside the fiber emission band should be 0, which is indicated by the red line.

### 3.B Linearity

**Figure 10:**
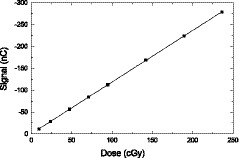
The detector signal measured by the photomultiplier tube as a function of incident dose.

Figure 10 shows the measured signal from the fiber segment in the detector block as a function of dose at the field size $2.4\;\times \;2.4\;{\text{cm}}^{2}$. The solid line is the linear fit to those data points. The measurement uncertainty is smaller than the size of the data points. The signal is indeed linear in the dose range measured.

### 3.C X‐ray absorption efficiency

The measured AE was 35% at a 6‐MV beam and independent of the x‐ray field sizes used. The AE was also calculated using a Monte Carlo (MC) simulation with Geant4, which gives a value of 35.8 ± 0.7%. The same 6 MV x‐ray spectrum used for calculation of DQE(0) in Section 2.7 was used in the simulation. The AE value from MC simulation agrees well with the measurement.

### 3.D Spatial resolution

**Figure 11:**
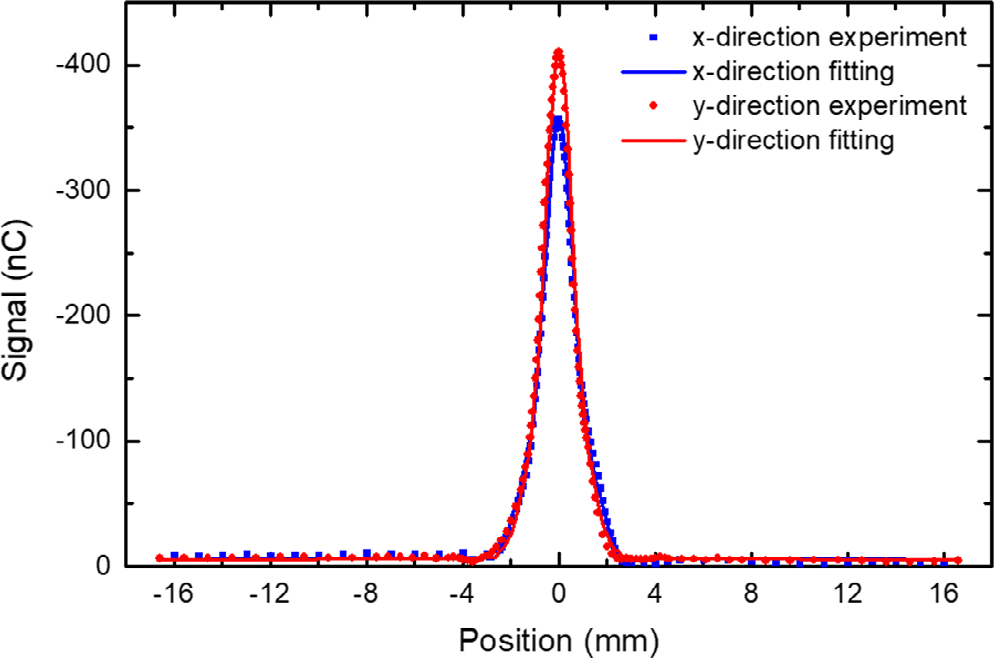
Line spread function of the detector in the x and y directions.

**Figure 12:**
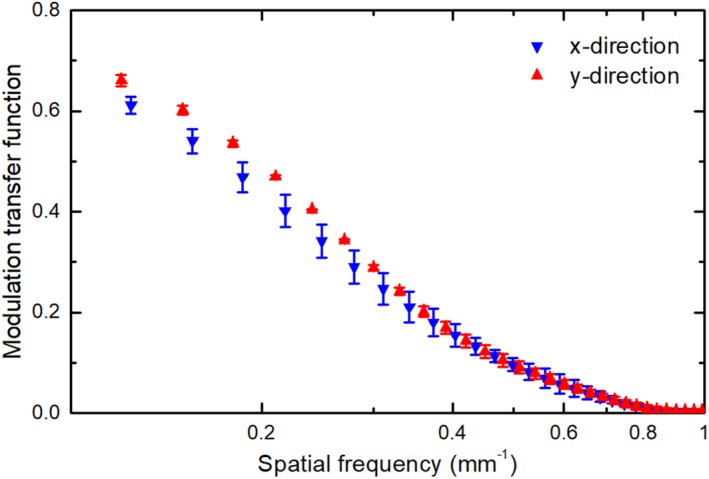
Modulation transfer function of the detector in the x and y directions. [Color figure can be viewed at http://www.wileyonlinelibrary.com]

Figure 11 shows the LSFs of the prototype scintillator in the x and y directions (denoted in Fig. 1). The full width at the half maximum of the LSFs is ∼1.4 mm. The LSF data were made symmetric before carrying out a Fourier transform to obtain the MTFs. Figure 12 shows the MTFs in the x and y directions. The measurement uncertainty was estimated based on two measurements on different days. The frequency at 50% modulation, $f_{50}$, in y direction is 0.2 ${\text{mm}}^{-1}$, which is comparable to the video‐based EPIDs, but lower than the current flat‐panel EPIDs with a $f_{50}$ of about 0.3–0.4 ${\text{mm}}^{-1}$.^35^, ^36^ The $f_{50}$ in the x direction is 0.17 ${\text{mm}}^{-1}$, not as high as that in the y direction.

### 3.E Antiscatter property

**Figure 13:**
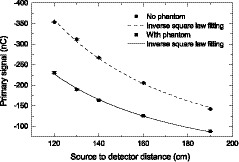
Detector signals due to the primary beam with and without phantom as a function of source to detector distance.

The primary signal of the prototype scintillator at different AGs were obtained by extrapolating the signal to field size zero, as shown in Fig. 8. At each AG, the field size was varied between $3\;\times \;3\;{\text{cm}}^{2}$ and $30\;\times \;30\;{\text{cm}}^{2}$. Figure 13 shows that the primary signals with and without phantom follow the inverse square law well, as indicated by the curve fitting results. Ratios of the primary signals in the presence of the phantom to those without the phantom, $R_{0}$, at different AGs are listed in Table 2. $R_{0}$ does not change with the AG in the range from 15 cm to 75 cm, which is expected and further validates the primary signals extrapolated, and it has a value of about 0.30. The $R_{0}$ at 5 cm AG is 0.32, higher than that at other AGs. This is probably due to that at 5 cm AG in the presence of the phantom, the secondary electrons produced in the phantom are able to travel to the prototype and excite optical photons in the scintillating fiber.

**Table 2:** Ratios ($R_{0}$) of the primary signals in the presence of the phantom to those without the phantom at different air gaps.

Air gap (cm)	$R_{0}$
5	0.322
15	0.300
25	0.302
45	0.301
75	0.304

**Figure 14:**
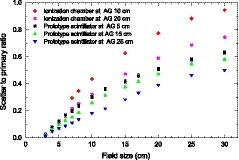
Scatter to primary ratios as a function of the field size at different air gaps for the prototype scintillator and ionization chamber detector. [Color figure can be viewed at http://www.wileyonlinelibrary.com]

Figure 14 shows the SPR as a function of field size at different AGs. The results from the prototype scintillator are compared to that based on the ionization chamber published earlier.^28^ The SPR decreases with the increase in the AG and increases with the field size for both types of detectors. For the same AG and field size, the SPR of the prototype scintillator is always lower than that of the ionization chamber detector. For the prototype scintillator, the SPR at 10 cm AG should be between the SPRs at 5 cm and 15 cm. At 10 cm AG and 20 cm × 20 cm field size, the SPR of the prototype scintillator is about 30% lower than that of the ionization chamber detector.

### 3.F Sensitivity of the detector

It was determined that 95.8% of the incident beam was attenuated by the slit object. Considering the field size used for the measurement, the dose delivered per MU LINAC output was 0.041 cGy to water at the ISO, which is less than two LINAC pulses.

The $\left\langle N_{\mathrm{ph}}\right\rangle$ can be calculated as $3.52\;\times \;10^{6}$ and $\sigma_{\mathrm{ph}}\;=\;2.69\;\times \;10^{4}$. The rms noise $\sigma_{\mathrm{ph}}$, which includes the x‐ray quantum and absorption noises at the dose 0.041 cGy, is much larger than the electronic noise per pixel (typically ∼2000e) of a conventional flat‐panel imager.^37^ Thus, the proposed detector using AMFPI for image readout would be quantum noise limited down to very low doses (∼a couple of radiation pulses of the LINAC).

### 3.G DQE(0)

**Figure 15:**
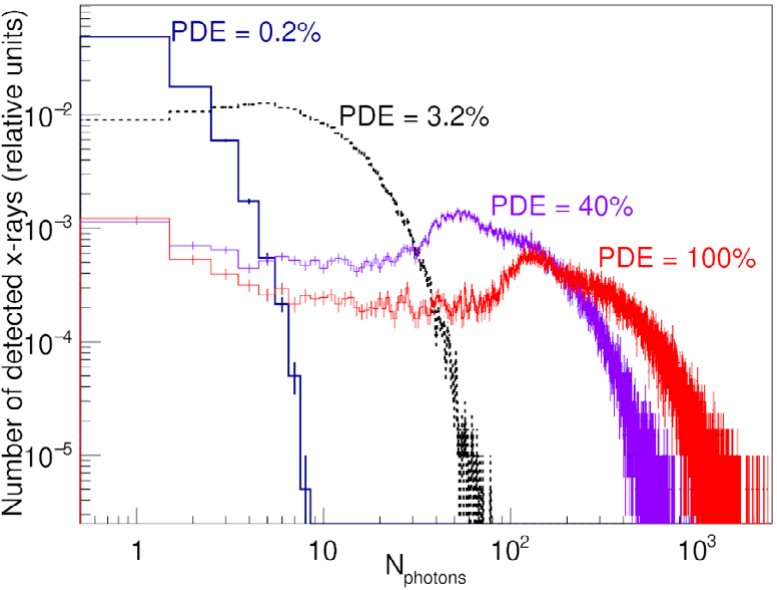
Distributions of number of optical photons detected by AMFPI per incident x‐ray per pixel for various value of optical photon detection efficiency of AMFPI.

Figure 15 shows the distribution of number of optical photons detected by AMFPI per incident x‐ray per pixel for various levels of PDE. The DQE(0) was calculated from the results of the simulation output using Eqs. 12 and 13. Sufficient number of incident x rays has been simulated to provide a statistical uncertainty in DQE(0) of ∼0.2%.

Table 3 lists DQE(0) for 6 MV simulated for various PDE levels. The PDE of an AMFPI used in conventional EPIDs is typically 50%,^2^ however, the actual value will depend on the effectiveness of the optical coupling between the AMFPI and the proposed detector. As shown in Table 3, the DQE(0) of the proposed detector with PDE≥ 3.2% is about an order of magnitude higher than that of current clinical EPIDs^2^ and ∼ 50% lower than that of the CdWO4‐based detector proposed recently by Star‐Lack et al.^9^

**Table 3:** DQE(0) of the proposed detector for various photon detection efficiency (PDE) levels.

PDE(%)	100	40	3.2	0.2
DQE(0) at 6 MV (%)	11.54	11.52	10.95	5.69

### 3.H Proof‐of‐concept image

**Figure 16:**
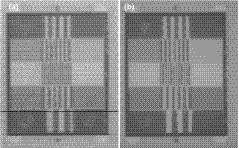
Proof‐of‐concept image of a PIPSPro QA‐3V phantom taken with the prototype detector (a) and an image of the same phantom taken with a clinical EPID system (b) at 6 MV. The imaging doses to the phantom used in both cases are ∼0.5 cGy or less (see text).

Figure 16(a) shows a single frame image of the PIPSPro phantom taken with the prototype detector at 6 MV using the estimated imaging dose to the phantom of less than 0.5 cGy. We note that this is a proof‐of‐concept image only. We have also included in Fig. 16(b) an image of the same phantom taken with a clinical EPID system (iViewGT, Elekta, UK) at 6 MV using 1 MU or a dose to the phantom of ∼0.5 cGy. As seen in Fig. 16(a), there are some artifacts in the proof‐of‐concept image, including two dark lines (due to the dead pixels in the AMFPI) and a background image of the scintillating fibers (due to an imperfect gain and offset correction).

## 4 Discussion

In this paper, we have proposed a new MV x‐ray detector and measured the AE, MTF, antiscatter property, and sensitivity of a prototype scintillator. The DQE(0) of the proposed detector was also calculated. We found the DQE(0) of the proposed detector can be about ten times higher than that of the current EPIDs used in most clinics. The proposed detector is, however, heavier than the current. The density of the proposed detector is 5.4 g/cm${}^{3}$ with the scintillator fill factor of 48%. For a detector with an area of 43 cm × 43 cm and a thickness of 2 cm, the weight of the detector without AMFPI is about 20 kg, which is not excessive as compared to that of current EPIDs.

The measured spatial resolution is lower in x direction than that in the y direction (Fig. 12). This is due to the optical epoxy used to glue the lead and fibers. The BC‐600 optical epoxy is highly transparent. The optical transmission through a 0.125‐mm‐thick layer is greater than 98% for wavelength above 400 nm according to the manufacturer. Since in the x direction there is a channel of epoxy (0.20 mm long in x direction) connecting the fibers (Fig. 1), cross‐talk is possible between fibers in x direction which would lower the resolution in x direction. The spatial resolution of the prototype scintillator is not as good as the current flat‐panel–based EPIDs^27^ and can be improved. It has been shown that the spatial resolution of a thick and high QE detector is determined by both the range of energetic electrons produced by x rays interacting with the detector and the reabsorption of x rays scattered within the detector.^35^ The large diameter and low density of the fiber deteriorate the resolution though the spacing lead helps shorten the range of the secondary electrons. Using fibers with smaller diameter should improve the spatial resolution.

**Figure 17:**
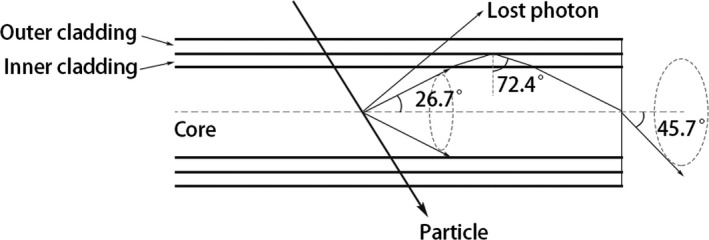
Diagram showing the optical photons that are excited in the scintillating fiber and able to exit the fiber.

For the scintillating fiber used, it can be calculated that only those photons with an incident angle equal to or greater than 72.4${}^{\circ }$ at the interface between the inner and outer claddings will be able to exit the fiber (Fig. 17), which accounts for only 5.3% of the total optical photons emitted in the fiber. Of these 5.3% photons, some will be lost due to the attenuation in the optical fiber and reflection at the interfaces. If the fiber wall could be coated with a reflective layer, not only the image signal would be improved but also the cross talk between fibers in the x direction would be eliminated.

It should be noted that the scintillating fibers in the prototype detector are not focused toward the source except the center fibers. The resolution would be slightly worse at the edge of the detector as compared to the center. For a typical source‐to‐detector‐surface distance of 159 cm, an unfocused detector of 2 cm thick would result in a geometrical blur of up to 0.37 mm for an imaging field size of 26 cm  × 26 cm at the isocenter, which is acceptable for MV imaging.

It is well understood that scattered x rays are detrimental to the image quality of an x‐ray imaging system. In general, there are three sources, from which scattered x rays are generated in MV x‐ray imaging in radiotherapy: LINAC head, patient (phantom), and detector itself. Scatter signal from all three sources were included in the measured SPR in this study. Our results show that the proposed detector can suppress the scatter signal. This is due to the use of lead as the spacing material. Lead not only helps improve the absorption of primary x rays and thus increase the QE of the detector but also acts like an antiscatter grid to some degree by blocking scattered x rays from producing signals in the detector. This is different from scatter correction methods that are based on scatter estimation and image processing to handle scatter after scattered x rays have produced signals in the detector.^38^

The image of the phantom taken with the prototype detector [Fig. 16(a)] is a proof‐of‐concept image only. There are also some artifacts that are usually absent in clinical EPID images^39^ due to the reasons mentioned earlier. In addition, the optical coupling between the prototype and AMFPI was not optimized: the surfaces of the prototypes were not polished and the gap between the bottom surface of the detector and the AMFPI was filled with air (not optical grease). Thus, there could be a significant optical photon loss in the interfaces between the prototype and the AMFPI. As demonstrated by our Monte Carlo simulation, the DQE is significantly dependent on the PDE of the AMFPI. Further investigation is needed to improve the image quality of the prototype. We note here that the MTF and DQE (0) were not measured using the prototype detector with the AMFPI readout. The MTF of the actual prototype detector could be slightly worse than the measured using the prototype scintillator due to a potential light spread at the interface between the scintillator and AMFPI. The DQE(0) of the actual prototype detector could also be lower than the calculated maximum due to light lost at the interface between the scintillator and AMFPI. However, the effect of both of these issues could be minimized by optimizing the optical coupling between the scintillator and AMFPI.

In this work, we have concentrated on the imaging application of the proposed detector. It is possible that the proposed detector could be used for dosimetric applications. However, there are challenges for dosimetric applications since the proposed detector is not water equivalent and is less sensitive to scattered radiation.

## 5 Conclusions

Properties of a proposed new high QE MV x‐ray detector for IGRT applications were evaluated using both experiments and Monte Carlo simulation. This detector responds linearly to the incident radiation dose. It can have a DQE(0) of 11.5% at 2 cm thickness, which is about an order of magnitude higher than that of current EPIDs. The resolution of the prototype scintillator is comparable to that of the video‐based EPIDs, but lower than that of flat‐panel–based EPIDs. The resolution can be improved by optimization of the design parameters. Due to the use of lead as the septal material between scintillating fibers, the new detector also has antiscatter property, which will help improve the signal‐to‐noise ratio of the image. As scintillating light yield is high, this detector is quantum noise limited at a very low dose, that is, a couple of LINAC pulses. Further investigation to improve the detector performance is warranted.

## Conflict of Interest

The authors have no conflict of interest to disclose.
